# A cytochrome P450 CYP71 enzyme expressed in *Sorghum bicolor* root hair cells participates in the biosynthesis of the benzoquinone allelochemical sorgoleone

**DOI:** 10.1111/nph.15037

**Published:** 2018-02-20

**Authors:** Zhiqiang Pan, Scott R. Baerson, Mei Wang, Joanna Bajsa‐Hirschel, Agnes M. Rimando, Xiaoqiang Wang, N. P. Dhammika Nanayakkara, Brice P. Noonan, Michael E. Fromm, Franck E. Dayan, Ikhlas A. Khan, Stephen O. Duke

**Affiliations:** ^1^ US Department of Agriculture Agricultural Research Service Natural Products Utilization Research Unit University MS 38677 USA; ^2^ National Center for Natural Products Research School of Pharmacy University of Mississippi University MS 38677 USA; ^3^ Department of Biological Sciences University of North Texas Denton TX 76203 USA; ^4^ Department of Biology University of Mississippi University MS 38677 USA; ^5^ Epicrop Technologies Inc. 5701 N. 58th Street, Suite 1 Lincoln NE 68507 USA

**Keywords:** allelochemical, CYP71AM1, cytochrome P450 monooxygenase, *Sorghum bicolor*, sorgoleone

## Abstract

- Sorgoleone, a major component of the hydrophobic root exudates of *Sorghum* spp., is probably responsible for many of the allelopathic properties attributed to members of this genus. Much of the biosynthetic pathway for this compound has been elucidated, with the exception of the enzyme responsible for the catalysis of the addition of two hydroxyl groups to the resorcinol ring.
- A library prepared from isolated *Sorghum bicolor* root hair cells was first mined for P450‐like sequences, which were then analyzed by quantitative reverse transcription‐polymerase chain reaction (RT‐qPCR) to identify those preferentially expressed in root hairs. Full‐length open reading frames for each candidate were generated, and then analyzed biochemically using both a yeast expression system and transient expression in *Nicotiana benthamiana* leaves. RNA interference (RNAi)‐mediated repression in transgenic *S. bicolor* was used to confirm the roles of these candidates in the biosynthesis of sorgoleone *in planta*.
- A P450 enzyme, designated CYP71AM1, was found to be capable of catalyzing the formation of dihydrosorgoleone using 5‐pentadecatrienyl resorcinol‐3‐methyl ether as substrate, as determined by gas chromatography‐mass spectroscopy (GC‐MS). RNAi‐mediated repression of CYP71AM1 in *S. bicolor* resulted in decreased sorgoleone contents in multiple independent transformant events.
- Our results strongly suggest that CYP71AM1 participates in the biosynthetic pathway of the allelochemical sorgoleone.

## Introduction

Allelopathy has been defined as the biosynthesis and release by an organism of one or more biologically active compounds, termed ‘allelochemicals’, that influence the growth, survival and reproduction of other organisms. In plants, allelopathic interactions are often characterized as a form of chemical warfare occurring between individuals competing for limited light, water and nutrient resources (Inderjit & Duke, 2003), and have also been proposed to have profound effects on the evolution of plant communities through the suppression of species susceptible to a given allelochemical (Schulz & Wieland, 1999). Furthermore, allelopathic compounds released by grain crop species are thought to play a significant role in the utility of cover crops and intercropping systems, where they act as weed suppressants. Allelopathic compounds have been characterized from a number of plants, including black walnut, wheat, rice and sorghum (Bertin *et al*., 2003; Duke, 2003; Inderjit & Duke, 2003).

Several *Sorghum* species have been reported to produce phytotoxins, which are exuded from their root systems into the rhizosphere and suppress the growth of competing species (Einhellig, 1996). For example, studies on the biologically active components of exudates from roots of *Sorghum bicolor* have demonstrated their role in the growth inhibition of lettuce seedlings (*Lactuca sativa*), as well as a number of important invasive weed species (Netzly & Butler, 1986). The major constituent of these exudates has been identified as 2‐hydroxy‐5‐methoxy‐3‐[(*Z*,*Z*)‐8′,11′,14′‐pentadecatriene]‐*p*‐benzoquinone, referred to as sorgoleone (Chang *et al*., 1986), which has been estimated to account for between *c*. 40% and 90% of the exudate material (w/w) in various accessions (Nimbal *et al*., 1996a; Czarnota *et al*., 2001; Dayan *et al*., 2009). The remaining exudate consists primarily of 4,6‐dimethoxy‐2‐((*Z*,*Z*)‐8′,11′,14′‐pentadecatriene)resorcinol (methoxy‐dihydrosorgoleone), and sorgoleone congeners differing in the length or degree of saturation of the aliphatic side chain and in the substitution pattern of the quinone ring (Erickson *et al*., 2001; Kagan *et al*., 2003; Rimando *et al*., 2003; Dayan *et al*., 2009). Sorgoleone biosynthesis is probably restricted to root hairs, which appear as cytoplasmically dense cells in sorghum, containing large osmiophilic globules deposited between the plasmalemma and cell wall, presumably associated with sorgoleone rhizosecretion (Czarnota *et al*., 2003a,b). The fact that sorgoleone acts as a potent broad‐spectrum inhibitor active against many agronomically important monocotyledonous and dicotyledonous weed species, and appears to affect multiple targets *in vivo* (Netzly & Butler, 1986; Einhellig & Souza, 1992; Nimbal *et al*., 1996b; Rimando *et al*., 1998; Czarnota *et al*., 2001; Bertin *et al*., 2003; Duke, 2003), may make it promising for development as a natural product alternative to synthetic herbicides (Duke, 2003).

**Figure 1:**
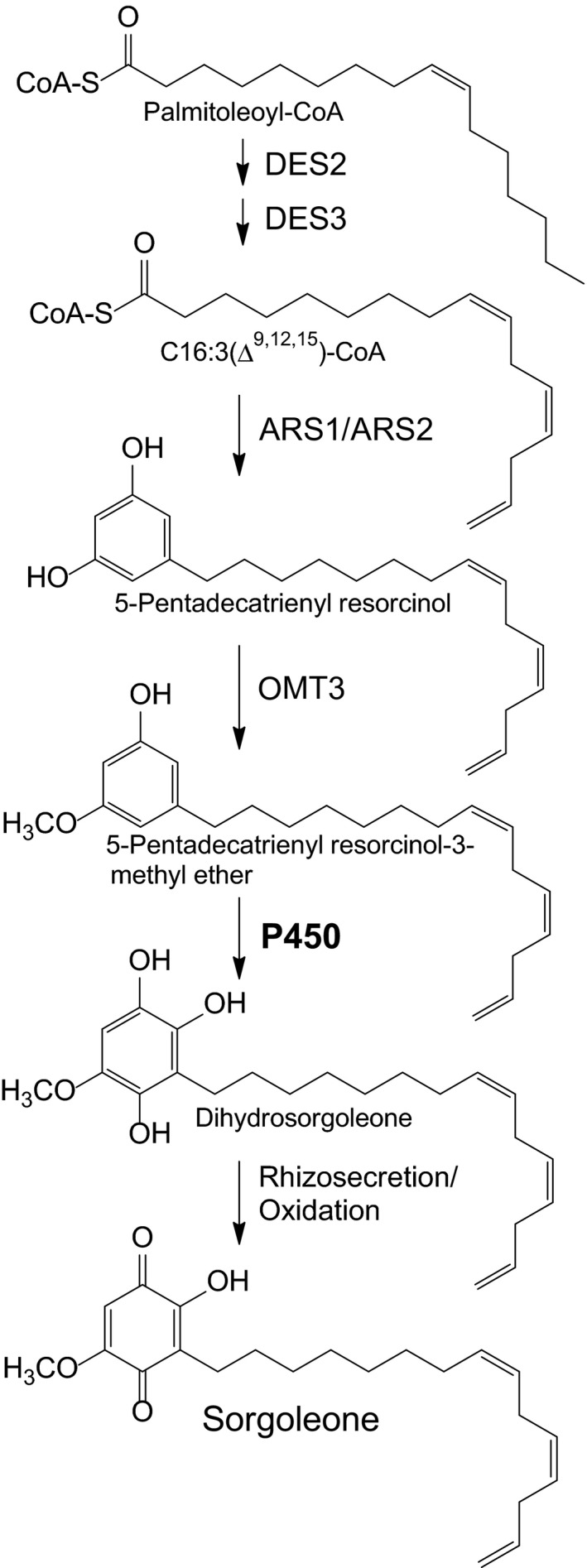
Proposed biosynthesis of sorgoleone from palmitoleoyl‐CoA. The involvement of one or more cytochrome P450s in the formation of dihydrosorgoleone from palmitoleoyl‐CoA is shown. Dihydrosorgoleone, the hydroquinone produced *in vivo*, is thought to undergo auto‐oxidation once secreted into the soil to yield sorgoleone, a more stable benzoquinone. ARS, alkylresorcinol synthase; DES, fatty acid desaturase; OMT,* O*‐methyltransferase; P450, cytochrome P450.

The herbicidal and allelopathic properties of sorgoleone make the isolation and characterization of the corresponding genes involved in sorgoleone biosynthesis highly desirable. For example, the manipulation of the biosynthetic pathway in sorghum, or the genetic modification of other plant species using these genes, could potentially provide advantages to growers by reducing their reliance on synthetic herbicide applications for weed control (Duke, 2003). To date, *S. bicolor* fatty acid desaturases (DES2, Pan *et al*., 2007; DES3, Yang *et al*., 2004; Pan *et al*., 2007), alkylresorcinol synthases (ARS1, ARS2; Cook *et al*., 2010) and a 5‐*n*‐alk(en)ylresorcinol‐utilizing *O*‐methyltransferase (OMT3; Baerson *et al*., 2008) have been identified which probably participate in the biosynthesis of sorgoleone *in vivo*. The final enzymatic step within the sorgoleone biosynthetic pathway leading to the formation of dihydrosorgoleone, which, on rhizosecretion, rapidly undergoes oxidation to yield sorgoleone (Fig. 1), has been proposed to be mediated by cytochrome P450s (Fate & Lynn, 1996; Dayan *et al*., 2003).

In an attempt to isolate genes encoding cytochrome P450 monooxygenases involved in the sorgoleone biosynthetic pathway, a library prepared from isolated *S. bicolor* (genotype BTx623) root hair cells was mined for the identification of candidate P450‐like sequences, which were then biochemically characterized using a yeast heterologous expression system. Herein, we describe the identification and functional characterization of a cytochrome P450 monooxygenase belonging to a subfamily of the plant‐specific CYP71 clan (designated CYP71AM1), capable of converting 5‐pentadecatrienyl resorcinol‐3‐methyl ether to dihydrosorgoleone.

## Materials and Methods

### Chemicals and plant materials

Standard laboratory reagents were purchased from Sigma Chemical Company (St Louis, MO, USA), Aldrich Chemical Co. (Milwaukee, WI, USA) and Fisher Scientific (Suwanee, GA, USA). Seeds of *Sorghum bicolor* (genotype BTx623) were purchased from Crosbyton Seed Co. (Crosbyton, TX, USA). Plant growth conditions were the same as described previously (Cook *et al*., 2010). Authentic sorgoleone standards were prepared from *S. bicolor* root systems, according to previously described methods (Dayan *et al*., 2003; Uddin *et al*., 2010). Mature leaves, stems and emerging panicles were harvested from *c*. 2‐month‐old, glasshouse‐grown, *S. bicolor* plants. Immature leaves and shoot apices were isolated from 8‐d‐old seedlings maintained in a growth chamber at 28°C, 16 h : 8 h, light : dark, 400 μmol m^−2^ s^−1^ light intensity. Total root systems and root hairs were isolated from 8‐d‐old seedlings grown using a capillary mat system (Czarnota *et al*., 2001; Yang *et al*., 2004). All tissues were collected and then flash‐frozen in liquid nitrogen, and kept at −80°C before RNA extraction. Root hairs were isolated according to the method of Bucher *et al*. (1997). *Nicotiana benthamiana* seeds were obtained from the USDA‐ARS, National Genetic Resources Program Germplasm Resources Information Network (GRIN), and plants were maintained in a growth chamber at 24°C, 16 h : 8 h, light : dark, 150 μmol m^−2^ s^−1^ light intensity.

### Identification of P450 sequences, RNA isolation and real‐time quantitative reverse transcription‐polymerase chain reaction (RT‐qPCR)

Database mining was performed using a library generated from isolated *S. bicolor* genotype BTx623 root hair cells, as described previously (Baerson *et al*., 2008; Cook *et al*., 2010). Cytochrome P450 sequences were identified by Blastn and tBlastn analysis (Altschul *et al*., 1997). Total RNAs for real‐time PCR experiments and cDNA cloning were isolated from flash‐frozen *S. bicolor* tissues using Trizol reagent (Invitrogen, Carlsbad, CA, USA), as described previously (Cook *et al*., 2010). RT‐qPCRs were performed in triplicate using a model 7300 sequence detection system (Applied Biosystems, Carlsbad, CA, USA), as described previously (Cook *et al*., 2010). Gene‐specific PCR primer pairs used for the 18S rRNA and three candidate P450s are listed in Supporting Information Table S1.

### Isolation of *S. bicolor* P450 clones

Full‐length cDNA clones encoding *S. bicolor* CYP71AM1 and CYP71AF1 were obtained from previously generated root hair contig consensus sequences (Baerson *et al*., 2008), and were used to direct 5′ and 3′ rapid amplification of cDNA ends (RACE) experiments with the BD Smart™ RACE cDNA Amplification Kit (Takara Bio, Mountain View, CA, USA) according to the manufacturer's instructions. Full‐length cDNAs were then amplified with primer pairs complementary to the 5′ and 3′ untranslated regions (UTRs) identified in RACE experiments using PfuUtra DNA polymerase (Agilent, Santa Clara, CA, USA) with first‐strand cDNA generated from RNA extracted from *S. bicolor* genotype BTx623 root hair cells. Several independent isolates from each amplification were sequenced to ensure the authenticity of the open reading frames (ORFs).

The sequences reported in this study have been deposited in the GenBank database (accession nos. CYP71AM1, MG020489; CYP71AF1, MG020490).

### Chemical synthesis of 5‐pentadecatrienyl resorcinol‐3‐methyl ether substrate

For the preparation of 5‐(8*Z*,11*Z*)‐8,11,14‐pentadecatrienyl resorcinol‐3‐methyl ether, the identity was confirmed using both physical and spectroscopic methods, including ^1^H‐NMR, ^13^C‐NMR and gas chromatography quadrupole time‐of‐flight mass spectroscopy (GC‐QTOF‐MS), as indicated below. Cashew nutshell liquid (Cardolite NC‐60; Cardolite Corp., Newark, NJ, USA) (115 g) was chromatographed over silica gel (1 kg) and eluted with hexanes : ethylacetate (95 : 5), which initially yielded a less polar fraction (78 g). Further elution with the same solvent yielded a fraction rich (> 95%) in 5‐(8*Z*,11*Z*)‐8,11,14‐pentadecatrienyl resorcinol (10.5 g). Spectroscopic data (^1^H‐NMR and ^13^C‐NMR) of this fraction agreed with those previously reported for this compound (Alvarenga *et al*., 2016).

A mixture of 5‐(8*Z*,11*Z*)‐8,11,14‐pentadecatrienyl resorcinol (8 g, 25 mmol – obtained from the procedure described above), methyl iodide (3.6 g, 25 mmol) and potassium carbonate (5 g, 36 mmol) in acetone (125 ml) was stirred at room temperature for 24 h. The reaction mixture was then filtered, and the filtrate was evaporated under vacuum. The oily product obtained was then chromatographed over silica gel and eluted with hexanes : ethylacetate (95 : 5), which initially yielded a dimethylated product. Further elution with the same solvent yielded 5‐(8*Z*,11*Z*)‐8,11,14‐pentadecatrienyl resorcinol‐3‐methyl ether (1.2 g). The characterization data for 5‐(8*Z*,11*Z*)‐8,11,14‐pentadecatrienyl resorcinol‐3‐methyl ether are provided below.

#### ^1^H‐NMR

(CDCl_3_, 500 MHz) δ 6.36 (1H, brs, H‐4), 6.30 (1H, brs, H‐6), 6.27 (1H, t, *J* = 2.1 Hz, H‐2), 5.86 1H (1H, m, H‐14′), 5.50–5.34 (4H, m, 8′, 9′, 11′ and H‐12′), 5.09 (1H, dd, *J* = 17.1, 1.5 Hz, H‐15′), 5.02 (1H, dd, *J* = 17.5, 1.5 Hz, H‐15′), 3.79 (3H, s, OCH_3_), 2.86 (2H, t, *J* = 5.9 Hz, H‐10′), 2.82 (2H, t, *J* = 6.3 Hz, H‐13′), 2.54 (2H, t, *J* = 7.5 Hz, H‐1′), 2.08 (2H, q, *J* = 6.7 Hz, H‐7′), 1.61 (2H, m, H‐2′), 1.44–1.29 (8H, m, H‐3′ to H‐6′).

#### ^13^C‐NMR

(CDCl_3_, 125 MHz) δ 160.7 (C‐1), 156.5 (C‐3), 145.7 (C‐5), 136.9 (C‐14′), 130.4 (C‐8′), 129.3 (C‐11′), 127.6 (C‐9′), 126.8 (C‐12′), 114.7 (C‐15′), 108.0 (C‐6), 106.8 (C‐4), 98.7 (C‐2), 55.3 (OCH_3_), 36.1 (C‐1′), 31.6 (C‐13′), 31.2 (C‐2′), 29.7, 29.4, 29.3, 29.2 (C‐3′ to C‐6′), 27.3 (C‐10′), 25.6 (C‐7′).

#### GC‐QTOF‐MS

[M]+ *m/z* 328.2393 (calculated for C_22_H_32_O_2_, 328.2402).

### Heterologous expression of recombinant cytochrome P450s in *Saccharomyces cerevisiae*

For heterologous expression in yeast, complete ORFs were amplified using PfuTurbo DNA polymerase (Agilent) and cloned in the pYeDP60 vector (Pompon *et al*., 1996) using the *Kpn*I and *Eco*RI restriction sites, yielding the plasmids pYeG12 (for CYP71AM1) and pYeF08 (for CYP71AF1). The sequence‐verified constructs were then transformed into the *S. cerevisiae* WAT11 strain (Pompon *et al*., 1996) using the lithium acetate procedure (Burke *et al*., 2000). For functional analysis of recombinant P450 enzymes, selected transformants were grown in SGI medium according to Pompon *et al*. (1996) and transferred to galactose‐containing induction medium containing 0.5% (v/v) tergitol Nonidet P40 (Sigma), a nonionic, nondenaturing detergent for the solubilization of the substrate. Substrate (5‐pentadecatrienyl resorcinol‐3‐methyl ether) was added to a final concentration of 0.2 mM. Cells were allowed to continue growth at 28°C for 16 h, and then harvested by centrifugation at 1500 ***g*** for 5 min. The cell pellet was then washed with 10 ml of 10 mM K_3_PO_4_ buffer, pH 7.5. Cells were then treated for 10 min in a Branson ultrasonic water bath (Danbury, CT, USA) with 10 ml of methanol. The mixture was clarified by centrifugation at 1000 ***g*** for 10 min. The methanol phase was recovered and dried under a stream of nitrogen. The dried extracts were treated with 100 μl of *N*,*N*‐bis(trimethylsilyl)trifluoroacetamide (BSTFA) in an oven at 125°C for 1 h. The treated extracts were then clarified by centrifugation at 14 000 ***g*** for 1 min, and the clear upper phase was recovered for GC‐MS analysis.

GC‐MS analysis was performed on an Agilent 7890 GC instrument equipped with an Agilent 5975C mass‐specific detector. An Agilent J&W HP‐5 capillary column (30 m, 0.25 mm ID, 0.25 μm film thickness) was used with helium as the carrier gas at a flow rate of 1 ml min^−1^ under the following oven conditions: an initial oven temperature of 120°C for 2 min, a ramp of 20°C min^–1^ to a final temperature of 300°C and held for 18 min. The injector temperature was 280°C. The split ratio was set to 10 : 1. One microliter aliquots of BSTFA‐derivatized extracts were injected.

The full‐scan mass spectra were recorded from 40 to 650 amu with the electron ionization (EI) source at 70 eV. The MS transfer line was kept at 280°C. The MS source temperature and quadrupole temperature were 230°C and 150°C, respectively.

### Transient expression of CYP71AM1 in *N. benthamiana*

The ORF of CYP71AM1 was subcloned into the shuttle vector pART7 (Gleave, 1992). The CYP71AM1::Tocs sequence was amplified with PfuTurbo polymerase using the primers ZP769 and ZP823 (Table S1). The 2x35S promoter was amplified from pTF101.1 using the primers ZP812 and ZP768 (Table S1). The 2x35S promoter and CYP71AM1::Tocs fragments were joined by fusion PCR using the primers ZP812 and ZP823 (Table S1) and PfuUltra HS DNA polymerase (Agilent) to generate the 2x35S promoter::CYP71AM1::Tocs terminator expression cassette. The expression cassette was then cloned into pLH7000 (Hausmann & Toepfer, 1999) using flanking *Sfi*I sites to generate the binary expression vector pLHG. The final expression cassette was verified by DNA sequence analysis.

The pLHG construct was transformed into the *Agrobacterium tumefaciens* strain EHA105. To improve transgene expression by suppression of post‐transcriptional gene silencing, the ORF of the p19 suppressor (Voinnet *et al*., 2003) was synthesized and inserted into the pCB404 binary vector (Rimando *et al*., 2012), resulting in pCB404‐P19, which was then mobilized into EHA105. Four to 5‐wk‐old *N. benthamiana* leaves were infiltrated with the strain expressing CYP71AM1 in combination with the strain expressing the suppressor of silencing, p19, in a ratio of 3 : 1, by injecting into the lower leaf epidermis using a 1‐ml syringe under gentle pressure as described previously (D'Aoust *et al*., 2008). Plants were grown and maintained in a growth chamber under a 16 h : 8 h, light : dark cycle at 25°C, 150 μmol m^−2^ s^−1^ light intensity.

Microsomes were prepared according to the methods described by Porchia *et al*. (2002). To monitor CYP71AM1 activity, microsomes (300 μg of protein) were incubated in a total volume of 300 μl containing 300 μM 5‐pentadecatrienyl resorcinol‐3‐methyl ether, 1 mM NADPH, 1 mM glucose 6‐phosphate, 0.5 U glucose 6‐phosphate dehydrogenase (Sigma) and 0.5% (v/v) tergitol Nonidet P40 at 28°C for 3 h. The reaction mixture was lyophilized overnight. The lyophilized sample was extracted with 100 μl of methanol by vortexing and sonication (Branson ultrasonic laboratory bath, model 2510) for 2 min. The sample was then centrifuged at 15 000 ***g*** at room temperature for 10 min. The methanol extract was collected, and the pellet was extracted a second time with 100 μl of methanol as described above. The supernatants were combined and dried under a stream of nitrogen. BSTFA was added to the dried sample to achieve a final concentration of 50 mg ml^−1^. The solution was heated at 100°C for 30 min, and then cooled to room temperature. The silylated extract was then analyzed by GC‐MS (Agilent 5793 mass‐selective detector) using a J&W DB‐5 capillary column (0.25 mm internal diameter, 0.25 mm film thickness, 30 m length; Agilent Technologies, Foster City, CA, USA). The GC temperature program was initially set at 210°C, and then raised to 300°C at a rate of 6°C min^−1^, and held at this temperature for 2 min. The carrier gas was ultrahigh purity helium, at a flow rate of 1.0 ml min^−1^. The inlet (splitless), GC interface and ion chamber temperatures were 250, 250 and 230°C, respectively. The sample injection volume used was 2.0 μl.

### Construction of binary vector for RNA interference (RNAi)‐mediated repression and sorghum transformation

The binary vector used for RNAi‐mediated suppression of *CYP71AM1* was prepared according to the methods described previously by Cook *et al*. (2010). The target region (480 bp) spanning contiguous portions of the CYP71AM1 3′ coding sequence and UTR was cloned in both sense and antisense orientation, separated by a 1.13‐kb intron spacer derived from the FAD2 gene of Arabidopsis (Okuley *et al*., 1994). The generation of transgenic *S. bicolor* events and the chemical analysis of sorgoleone contents in *S. bicolor* seedling roots were performed according to the methods described previously (Howe *et al*., 2006; Cook *et al*., 2010). Transgenic (T‐DNA ‘+’) and null segregant (T‐DNA ‘−’) individual seedlings in segregating T_1_ populations were identified using a neomycin phosphotransferase II enzyme‐linked immunosorbent assay (NPTII‐ELISA) kit (Agdia Inc., Elkhart, IN, USA).

### Phylogenetic analysis

Amino acid sequences of putative plant CYP71 family cytochrome P450s were retrieved from the National Center for Biotechnology Information (NCBI) nonredundant peptide sequence database by Blastp searches using default parameters (http://blast.ncbi.nlm.nih.gov). A candidate list was screened for redundancy and errors, and a final dataset was assembled containing 48 sequences, including the two *S. bicolor* sequences biochemically characterized in the present work. Multiple sequence alignments were constructed with Geneious v.4.6.2 (Biomatters Ltd, Auckland, New Zealand) employing the Blosum62 log‐odds probability matrix (Henikoff & Henikoff, 1993) and gap open and extension penalties of 12 and 3, respectively. As the resulting alignment possessed multiple regions of questionable homology, it was pruned of poorly aligned regions using the program Gblocks (Talavera & Castresana, 2007) on the Gblocks Server (http://molevol.cmima.csic.es/castresana/Gblocks_server.html) with both high (disallowing many contiguous nonconserved regions) and low (allowing smaller blocks, gaps within and less strict flanking regions) stringency settings.

Phylogenetic relationships among amino acid sequences were inferred for the three separate alignments using the Bayesian Markov chain Monte Carlo technique implemented in Mrbayes v.3.1 (Ronquist & Huelsenbeck, 2003). This analytical approach takes advantage of probabilistic models of amino acid substitution and has been shown to be robust to among‐site rate heterogeneity and branch length differences (Mar *et al*., 2005). Two separate analyses, each containing two independent searches, were run for each alignment (original, Gblocks high‐stringency, Gblocks low‐stringency) for 10^8^ generations, sampling every 2000. To incorporate the uncertainty in the appropriate amino acid substitution model, we used mixed priors with gamma‐distributed rate variation; posterior support for the Wagner model (Whelan & Goldman, 2001) was 1.0. From this posterior sample of trees from each analysis (*n *=* *5000), the first 1000 were discarded as burnin. The adequacy of this burnin was assessed by the examination of likelihood values of the cold chain for stationarity using Tracer v.1.4 (http://beast.bio.ed.ac.uk/tracer). Support for proposed relationships was assessed by examination of the bipartition posterior probability, the frequency of occurrence of a relationship, in the 16 000 pooled post‐burnin trees.

### Homology modeling and docking

The comparative modeling program Modeller (Fiser & Sali, 2003) was used to generate models for CYP71AM1, with the structures of human P450 17A1 (PDB ID: 4NKV) and P450 1A2 (PDB ID: 2HI4) used as templates in the structural modeling experiments. The three‐dimensional model of CYP71AM1 was obtained by optimal satisfaction of the spatial restraints derived from sequence alignments based on the Clustalx results and three‐dimensional structures. The heme structural model in CYP71AM1 was obtained by superimposing the CYP71AM1 model onto the human P450 17A1 structure containing a heme co‐factor. For molecular docking of the substrate 5‐pentadecatrienyl resorcinol‐3‐methyl ether to CYP71AM1, the automated docking program Autodock was used, and the structure of human P450 17A1 bound with inhibitor abiraterone was used as a reference. All structural models were analyzed and minor manual adjustments of the modeling solution were made using the graphics program Coot (Emsley & Cowtan, 2004).

## Results

### Identification of cytochrome P450‐like sequences preferentially expressed in *S. bicolor* root hair cells

**Figure 2:**
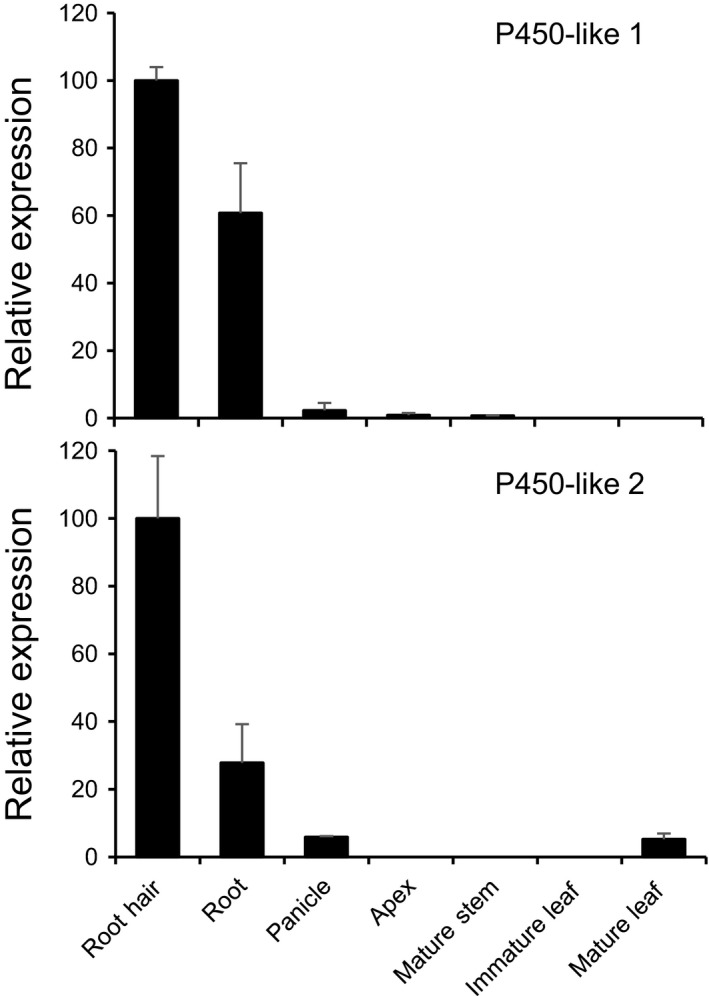
Relative expression of two P450‐like sequences identified in a library prepared from isolated *Sorghum bicolor* root hair cells. The relative expression levels were determined by quantitative reverse transcription‐polymerase chain reaction (RT‐qPCR) using gene‐specific primers. Data were normalized to an internal control (18S rRNA), and the ΔΔ*C*_T_ method was used to obtain the relative expression levels for each sequence, expressed as the mean ± SD from assays performed in triplicate.

One of the key enzymatic reactions in the biosynthetic pathway of sorgoleone is the dihydroxylation of the 5‐pentadecatrienyl resorcinol‐3‐methyl ether intermediate, probably mediated by P450 monooxygenases, which yields dihydrosorgoleone (Fig. 1). To isolate cytochrome P450 clones potentially involved in these hydroxylation reactions, we first mined a library prepared from isolated *S. bicolor* genotype BTx623 root hair cells (Baerson *et al*., 2008; Cook *et al*., 2010) for expressed P450‐like transcripts. From these analyses, 11 unique P450‐like sequences were identified. Given that sorgoleone biosynthesis occurs solely in root hairs (Czarnota *et al*., 2003a,b), the tissue‐specific expression patterns were then examined for all identified P450‐like sequences, by RT‐qPCR, to identify candidates expressed specifically or predominantly in this cell type, using cDNAs prepared from isolated root hairs, total root systems, developing panicles, stems, immature and fully expanded leaves, and shoot apices as templates. Based on these results, two P450‐like sequences preferentially expressed in root hairs were selected for further study (Fig. 2). The primers used in this experiment are listed in Table S1. Full‐length cDNA clones were then generated for both sequences using cDNA prepared from isolated root hair RNA. The predicted protein sequences derived from the two cDNAs (Fig. S1) exhibited all of the major conserved motifs typically found in plant cytochrome P450 monooxygenases, such as the proline‐rich region, the O_2_ binding site and the E‐R‐R triade located upstream of the heme‐binding cysteine motif (Bak *et al*., 2011). In addition, both predicted proteins corresponded to typical members of the plant‐specific CYP71 family, and P450‐like sequences 1 and 2 (Fig. 2) were designated *S. bicolor* CYP71AM1 and CYP71AF1, respectively. This nomenclature will therefore be used hereafter in reference to these sequences.

### Functional characterization of CYP71AM1 and CYP71AF1 in *S. cerevisiae*

A yeast heterologous expression system was used to investigate the activities of both CYP71AM1 and CYP71AF1. Two plasmids, pYeG12 carrying CYP71AM1 and pYeF08 carrying CYP71AF1, were constructed by inserting the full‐length ORFs into the yeast expression vector pYeDP60 (Pompon *et al*., 1996). The recombinant proteins were expressed in the *S. cerevisiae* WAT11 strain which co‐expresses the NADPH‐cytochrome P450 reductase gene from *Arabidopsis thaliana* (Urban *et al*., 1990; Pompon *et al*., 1996). CO difference spectra with characteristic peaks at 450 nm were obtained from microsomal fractions isolated from yeast cells expressing CYP71AM1 and CYP71AF1 (Fig. S2), indicating the presence of a functional cytochrome P450 in both cases.

**Figure 3:**
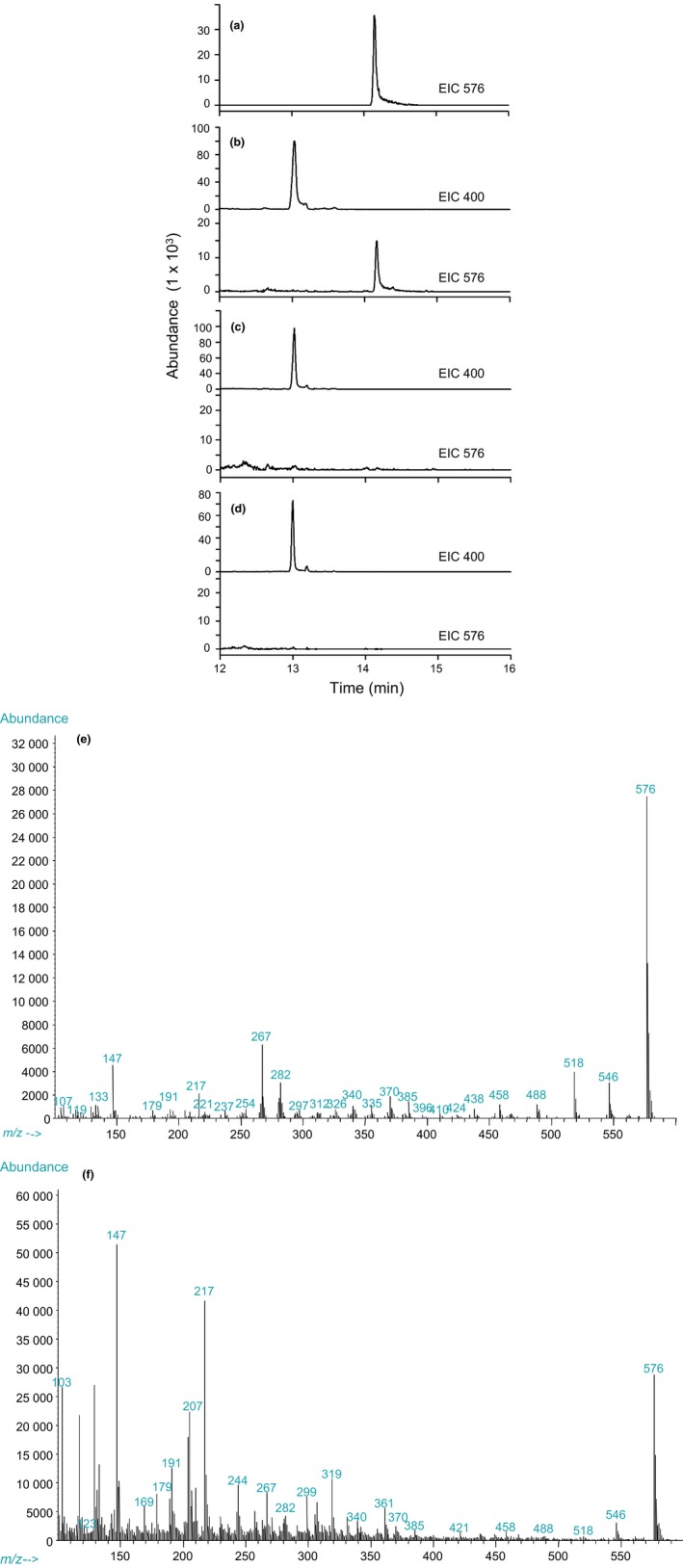
Gas chromatography‐mass spectroscopy (GC‐MS) analysis of extracts from yeast cultures. Extracted ion chromatograms (EICs) are shown detailing a single intense peak at 13.03 min for the trimethylsilyl (TMS) derivatized substrate (*m/z *=* *400) and at 14.16 min for dihydrosorgoleone (*m/z *=* *576). (a) The EIC for the ion *m/z* 576 generated from sorgoleone extracted from *Sorghum bicolor* root systems. (b) The EICs for the *m/z* 400 and 576 ions generated from yeast strains expressing *S. bicolor* CYP71AM1. (c) The EICs for the *m/z* 400 and 576 ions generated from yeast strains expressing *S. bicolor* CYP71AF1. (d) The EICs for the *m/z* 400 and 576 ions generated from (empty vector) control strains. (e, f) Mass spectra of TMS‐derivatized products (EIC576) corresponding to the peaks shown in a (e) and b (f).

In an effort to determine the potential hydroxylase activity of the recombinant proteins, *in vivo* enzymatic assays were conducted using yeast strains harboring either pYeG12 or pYeF08. Galactose‐induced transformed yeast cell cultures were incubated in the presence of the predicted physiological substrate 5‐pentadecatrienyl resorcinol‐3‐methyl ether, and reaction products were then analyzed by GC‐MS. Yeast strains harboring the pYeDP60 expression vector alone were also cultured in parallel as empty vector controls. As seen in Fig. 3, when substrate was provided to recombinant yeast strains expressing CYP71AM1 (Fig. 3b), a peak at 13.03 min, associated with 5‐pentadecatrienyl resorcinol‐3‐methyl ether, was observed, as well as the appearance of an additional peak at 14.16 min, which was identical to the peak observed in chromatograms generated from authentic dihydrosorgoleone (Fig. 3a), indicating that CYP71AM1 has dihydroxylation activity with the physiological substrate. Moreover, the ion fragments observed in the mass spectrum for the peak appearing at 14.16 min in the CYP71AM1‐containing assays (Fig. 3f) were consistent with the fragmentation of the dihydrosorgoleone standard (Fig. 3e). For strains expressing the *S. bicolor* CYP71AF1 enzyme, however, the substrate appeared to remain un‐metabolized following incubation (Fig. 3c), and the chromatograms obtained were similar to those observed for empty vector control strains (Fig. 3d). Taken together, these results indicate that CYP71AM1 is capable of hydroxylating 5‐pentadecatrienyl resorcinol‐3‐methyl ether at the ortho positions (C4 and C6) from the aliphatic chain, resulting in the conversion of the resorcinol into dihydrosorgoleone (a hydroquinone: 5‐methoxy‐3‐((8*Z*,11*Z*)‐pentadeca‐8,11,14‐trien‐1‐yl)benzene‐1,2,4‐triol), the direct precursor of sorgoleone (Fig. 1). As recombinant CYP71AF1 did not exhibit activity in the yeast expression system with this substrate, all subsequent experiments were focused on the analysis of CYP71AM1.

### Transient expression of CYP71AM1 in *N. benthamiana*

To examine activity in a plant system, a construct was prepared for transient expression of recombinant CYP71AM1 in *N. benthamiana* plants under the control of the cauliflower mosaic virus (CaMV) 35S promoter. *Nicotiana benthamiana* plants were co‐infiltrated with *A. tumefaciens* strains transformed with a CYP71AM1 expression vector and a vector for expression of the RNA silencing suppressor p19 protein (Voinnet *et al*., 2003), also driven by the 35S promoter. Empty vector *A. tumefaciens* strains were also prepared for use as negative controls. In addition, Blast searches were performed with the *N. benthamiana* genome v.1.0.1 draft (https://solgenomics.net) to identify potential endogenous CYP71AM1 homologs, and none were identified. The transient expression of *S. bicolor* CYP71AM1 in *N. benthamiana* was also confirmed by RT‐qPCR (Fig. S3).

**Figure 4:**
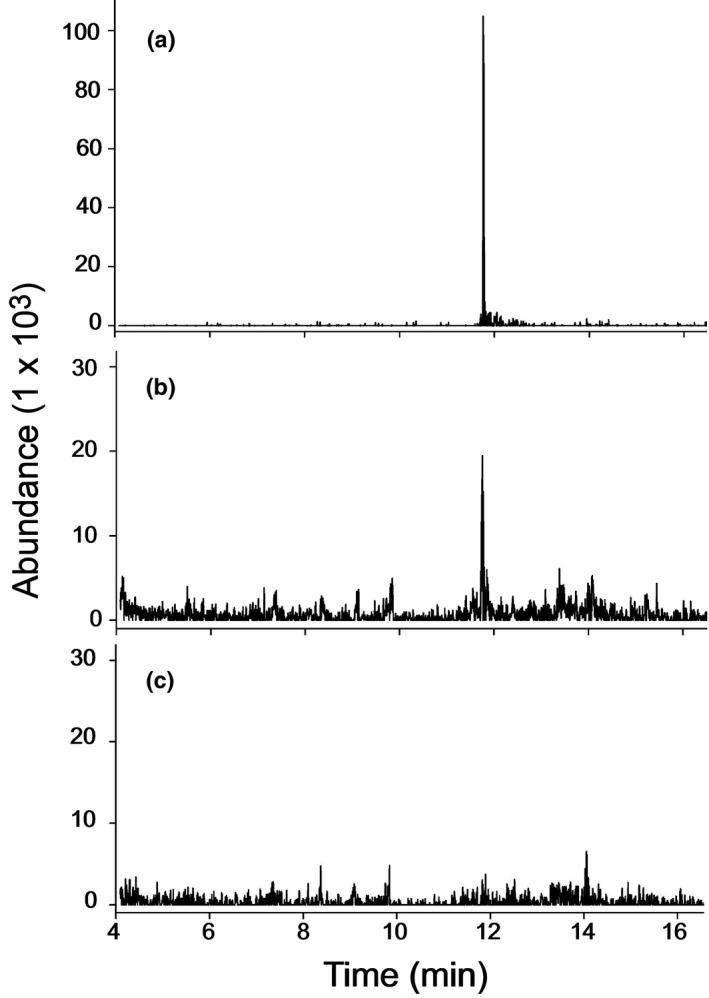
Enzyme activity detected in microsomes extracted following the transient expression of CYP71AM1 in *Nicotiana benthamiana*. (a) Total ion chromatogram of sorgoleone purified from *Sorghum bicolor* root systems. (b, c) Total ion chromatograms of methanol extracts from microsomes extracted from agroinfiltrated *N. benthamiana* leaves co‐expressing p19 and CYP71AM1 (b) or p19 and the empty T‐DNA vector (pLH7000) control (c).

When microsomes prepared from infiltrated *N. benthamiana* leaves expressing *S. bicolor* CYP71AM1 were incubated with the physiological substrate 5‐pentadecatrienyl resorcinol‐3‐methyl ether, dihydrosorgoleone was identified within the reaction mixtures (Fig. 4b), as evidenced by the presence of a peak observed in the chromatogram identical to that observed for the dihydrosorgoleone standard (Fig. 4a). In addition, dihydrosorgoleone was not detected in reaction mixtures when microsomes were prepared from tissues infiltrated with the control (empty vector) *A. tumefaciens* strain (Fig. 4c). Thus, these results demonstrate that the recombinant CYP71AM1 enzyme was expressed in an active form *in planta*, and corroborate the activity seen using the *S. cerevisiae* expression system (Fig. 3).

### Involvement of CYP71AM1 in sorgoleone biosynthesis in *S. bicolor* as revealed by RNAi‐mediated repression

**Figure 5:**
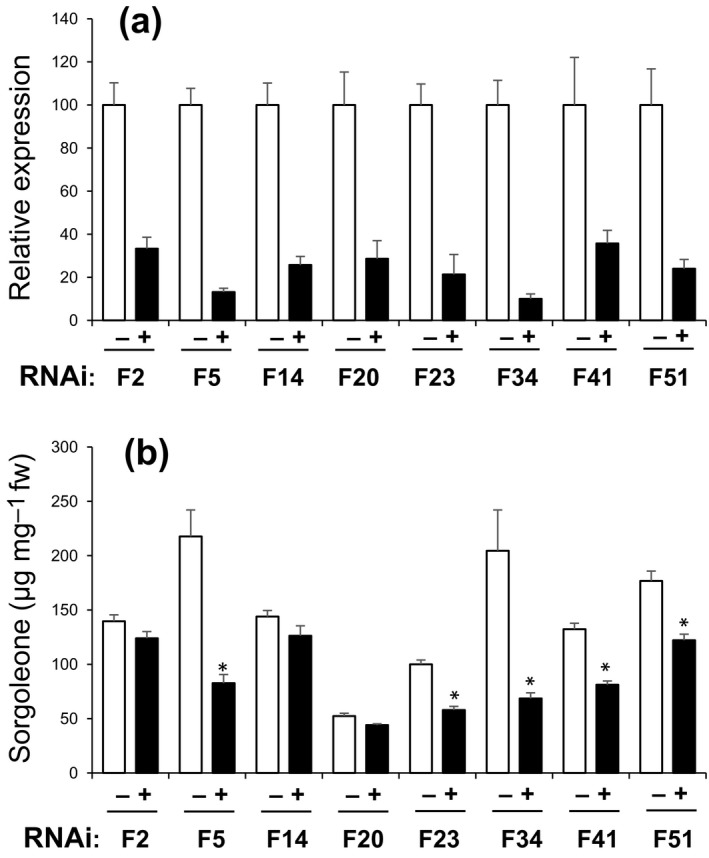
Analysis of *Sorghum bicolor* RNA interference (RNAi) transformant events. (a) Relative CYP71AM1 endogenous transcript levels in 10‐d‐old *S. bicolor* T‐DNA ‘+’ and T‐DNA ‘−’ seedlings were determined by quantitative reverse transcription‐polymerase chain reaction (RT‐qPCR) using gene‐specific primers. Data were normalized to an internal control (18S rRNA). (b) Sorgoleone levels were determined by gas chromatography‐mass spectroscopy (GC‐MS) analysis of root exudates prepared from 10‐d‐old T‐DNA ‘+’ and T‐DNA ‘−’ seedlings. Bars with asterisks above indicate significant differences in comparison with the corresponding T‐DNA ‘−’ plants as determined by Student's *t*‐test: *, *P *<* *0.05. Bars are SD for three biological replicates.

To further evaluate the potential role of this enzyme in sorgoleone biosynthesis, a hairpin RNA‐forming binary vector was constructed for targeted downregulation of *CYP71AM1* via RNAi (Fig. S4). The RNAi construct was used for stable transformation of *S. bicolor* genotype Tx430, and included a 480‐bp region corresponding to portions of the 3′ coding region and the UTR of *CYP71AM1*, which was cloned in both sense and antisense orientation, separated by a 1.13‐kb intron spacer derived from the FAD2 gene of Arabidopsis (Okuley *et al*., 1994). Eight independent *S. bicolor* transformant events were selected to evaluate the effects of *CYP71AM1* downregulation on sorgoleone biosynthesis *in planta*, using segregating T_1_ seedlings. We first pre‐screened individual 10‐d‐old seedlings from all eight events grown in perlite culture (Cook *et al*., 2010) for the presence of the T‐DNA using a nondestructive NPTII‐ELISA and, based on these results, pooled root tissue samples into T‐DNA ‘+’ or T‐DNA ‘−’ pools for each event. The same ‘+’ and ‘−’ samples from each event were subsequently used for RNA extraction and analysis of sorgoleone content levels by GC‐MS (Fig. 5).

The expression levels for endogenous *CYP71AM1* transcripts in ‘+’ and ‘−’ individuals were independently assayed for each event by RT‐qPCR using gene‐specific primers as shown in Fig. 5(a). In all events, *CYP71AM1* expression levels were substantially reduced in T‐DNA ‘+’ individuals relative to T‐DNA ‘−’, reflecting the successful downregulation of *CYP71AM1* in transformants. Reduction of *CYP71AM1* transcript accumulation in T‐DNA ‘+’ individuals ranged from *c*. 61% to 90% relative to corresponding T‐DNA ‘−’ individuals for each event, and complete loss of *CYP71AM1* expression was not observed for any event (Fig. 5a). Sorgoleone accumulation levels in five (out of eight) events were significantly reduced in T‐DNA ‘+’ relative to corresponding T‐DNA ‘−’ individuals from the same event (Fig. 5b), consistent with the hypothesis that CYP71AM1 represents the P450 enzyme participating in the final enzymatic step of the sorgoleone biosynthetic pathway in *S. bicolor*.

### Phylogenetic analysis

**Figure 6:**
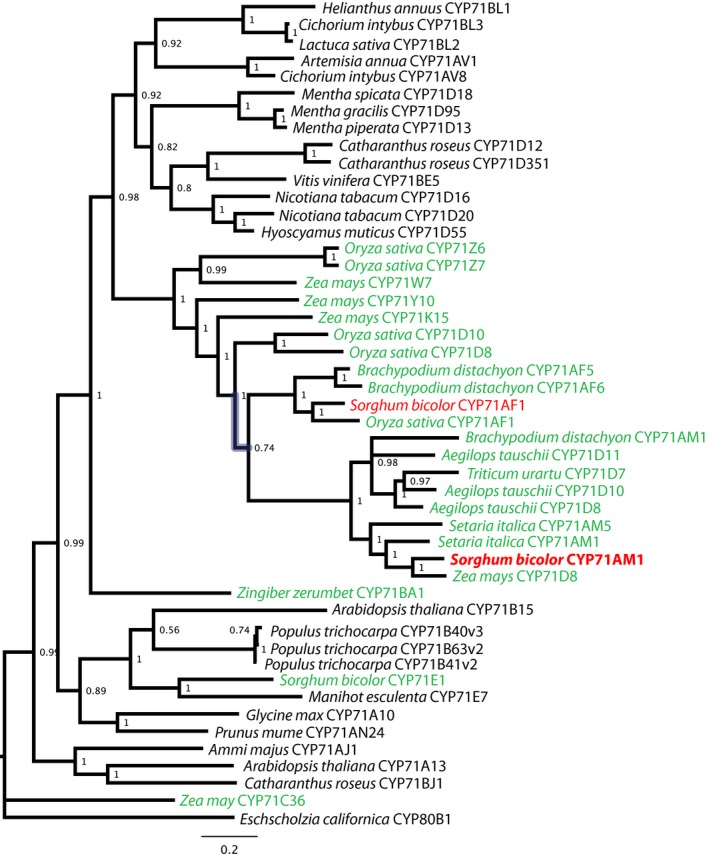
Phylogenetic relationships of CYP71 P450 family members inferred from Bayesian analysis of the full alignment of amino acid sequences. Numbers at the nodes indicate posterior probability and taxonomic names in colored font indicate monocots (green) and the focal species *Sorghum bicolor* (red); taxonomic names are followed by enzyme subfamily designations. GenBank accession numbers and known biochemical functions (where available) are provided in Supporting Information Table S2.

To examine the phylogenetic relationship between CYP71AM1 and other cytochrome P450 enzymes, a phylogenetic tree was constructed based on sequences of selected members of the plant‐specific CYP71 P450 family. Inferred relationships and statistical support differed little between that based on the complete alignment and those based on alignments in which poorly aligned regions had been pruned. All phylogenetic analyses, regardless of the extent to which poorly aligned regions were pruned, placed the *S. bicolor* CYP71AM1 protein sequence within a strongly supported (posterior probability = 1.0) clade comprising both CYP71AM and CYP71D subfamily members. It is important to take into account, however, that the CYP71AM subfamily is essentially an extension of CYP71D, created as a result of the large numbers of predicted sequences designated as CYP71D members within recently sequenced plant genomes (D. Nelson, pers. comm.). Thus, based on the current plant P450 nomenclature, CYP71AM/CYP71D can be considered as a single P450 subfamily that is highly differentiated from, but most similar to, CYP71AF, with *S. bicolor* CYP71AM1 being the only member which has been functionally characterized to date (Fig. 6).

### Docking analysis of substrate interactions

**Figure 7:**
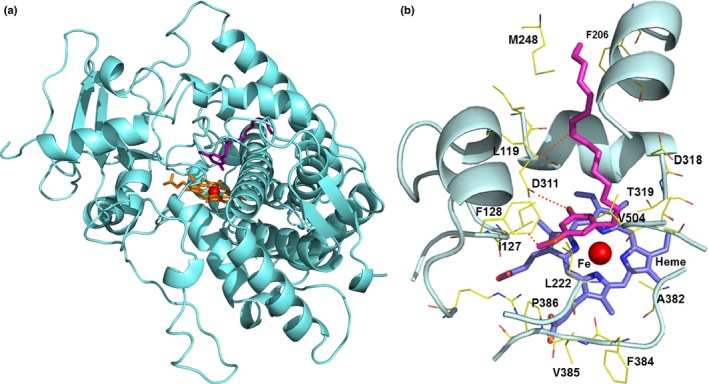
Molecular docking model for *Sorghum bicolor* CYP71AM1 with the proposed physiological substrate. (a) Ribbon diagram of the CYP71AM1 homology model docked with heme and the substrate 5‐pentadecatrienyl resorcinol‐3‐methyl ether. Both heme and substrate are shown as stick models in orange and magenta, respectively, and the heme iron is shown as a red sphere within the heme model. (b) Close‐up view of the substrate binding pocket derived from the CYP71AM1 model. The substrate is shown as a stick model in magenta. Selected protein residues within the binding pocket are labeled and shown as thin line models. Possible Van der Waals’ interactions and hydrogen bonds are indicated with red dotted lines.

As indicated above, CYP71AM1 catalyzes a unique reaction involving the addition of two hydroxyl groups to the ortho positions of the resorcinol ring from the aliphatic chain; therefore, a molecular docking approach was used to gain further insights into the potential reaction mechanism involved. For this purpose, a homology model for CYP71AM1 was first generated based on the published crystal structures of human P450 17A1 (PDB ID: 4NKV) and P450 1A2 (PDB ID: 2HI4). Similar to human P450 17A1 and other P450s for which structures have been reported, CYP71AM1 possesses a CYP fold containing a highly conserved structural core surrounding the heme group, including the heme‐binding loop on the proximal face of the heme with the conserved cysteine as fifth ligand to the heme iron, and the I helix containing a P450 signature motif for oxygen binding, with a conserved threonine T319 pointing towards the oxygen binding site (Fig. 7a).

The substrate binding pocket is located on the distal side of the heme group, and is relatively hydrophobic and formed from hydrophobic residues, for example, L119, I127, F128, F206, L222, M248, I310, A382, F384, V385, P386 and V504. In addition, the substrate 3‐methyl‐5‐pentadecatrienyl resorcinol was docked into the active site of the CYP71AM1 model structure (Fig. 7b). In the docking model, the long aliphatic chain of the substrate is positioned within the long access channel formed by the loops between helices B and C (the B–C loop) and helices F and G (the F–G loop). The substrate aromatic ring is positioned above the heme group with its C4 and C6 carbons situated proximal to the heme iron. Based on this model, residues L119, I127 and F128 in the B–C loop and L222 in the F–G loop may contribute to substrate binding and specificity. Interestingly, an acidic residue (D311) is positioned close to the heme group and may directly interact with the substrate 1‐OH and thereby play an important role in substrate specificity. D318 is also positioned within the substrate binding pocket and is relatively distal from the two hydroxylation sites, but may play a role in substrate binding. In the current docking model, the C4 site may be slightly preferred for hydroxylation; however, the environment and interactions for C4 and C6 appear to be quite similar (Fig. 7b).

## Discussion

The biosynthetic pathway for sorgoleone, an allelochemical produced by *Sorghum* spp. members, has been well established (Fate & Lynn, 1996; Dayan *et al*., 2003). A number of enzymes involved in the pathway have been identified and functionally characterized, including fatty acid desaturases (Pan *et al*., 2007), an *O*‐methyltransferase (Baerson *et al*., 2008) and alkylresorcinol synthases (Cook *et al*., 2010). It has been proposed that the final step of the enzymatic reactions within the biosynthetic pathway involves cytochrome P450 enzymes that mediate the dihydroxylation of 5‐pentadecatrienyl resorcinol‐3‐methyl ether to produce dihydrosorgoleone, which, when released into the soil, rapidly undergoes oxidation to yield the phytotoxic compound sorgoleone (Fig. 1). In this study, we identified a cytochrome P450 enzyme (CYP71AM1) which can catalyze dihydroxylation at the C4 and C6 positions of the resorcinolic intermediate when provided as substrate. To our knowledge, this represents the first report of a P450 enzyme capable of performing dihydroxylation on a resorcinol ring moiety.

Plant cytochrome P450 enzymes capable of catalyzing sequential hydroxylation reactions have been reported previously. For example, flavonoid‐3′,5′‐hydroxylases (CYP75As) from *Petunia hybrida* catalyze the stepwise hydroxylation of the B‐ring of flavonoid intermediates in the anthocyanin biosynthetic pathway (Holton *et al*., 1993). Ralston *et al*. (2001) demonstrated that 5‐*epi*‐aristolochene‐1,3‐dihydrolase (CYP71D20) from *Nicotiana tabacum* was capable of catalyzing both the C1 and C3 hydroxylations of 5‐*epi*‐aristolochene in a sequential manner (without releasing an intermediate), resulting in the formation of capsidiol. Here, we show that CYP71AM1 is able to convert 5‐pentadecatrienyl resorcinol‐3‐methyl ether to dihydrosorgoleone via hydroxylation at positions 4 and 6 of the resorcinol ring; however, whether this dihydroxylation occurs in a sequential manner remains unknown. Molecular docking studies indicate that the substrate is positioned within the relatively hydrophobic substrate binding pocket, with the aliphatic side chain positioned within a long hydrophobic access channel. Within the active site, the conserved threonine T319 residue from the P450 signature motif and I helix points towards the oxygen binding site. The acidic residue D311 may interact with the hydroxyl group of the substrate to play an important role in the determination of substrate specificity.

*CYP71AM1* transcripts were found to accumulate primarily in root hair cells in which sorgoleone biosynthesis is thought to be exclusively localized (Fig. 2; Czarnota *et al*., 2003a,b). Moreover, RNAi‐mediated interference experiments targeting *CYP71AM1* significantly reduced the accumulation of sorgoleone *in vivo* (Fig. 5), consistent with CYP71AM1 playing a direct role in sorgoleone biosynthesis. In our previous report, RNAi experiments targeting *ARS1* and *ARS2*, which encode the specialized type III polyketide synthase enzymes responsible for the generation of the 5‐pentadecatrienyl resorcinol pathway intermediate, resulted in the reduction of sorgoleone accumulation to levels below the limit of quantification of the GC‐MS analytical methods used (Cook *et al*., 2010). In the report by Cook *et al*. (2010), *ARS1* and *ARS2* were downregulated simultaneously because of their high degree of shared sequence identity, resulting in the nearly complete elimination of sorgoleone production. In the present experiments, in which we targeted the expression of *CYP71AM1* using RNAi, although sorgoleone production was significantly reduced in the majority of transgenic lines analyzed, it was not inhibited to nearly the same extent as seen when *ARS1* and *ARS2* were targeted. This may imply that the extent of RNAi‐mediated inhibition of *CYP71AM1* expression that was achieved was insufficient to inhibit sorgoleone production more severely, there could be additional CYP genes performing redundant functions within the *S. bicolor* genome, and ARS1 and ARS2 activities could be rate limiting or serve as pathway regulatory points, and this may not be the case for CYP71AM1, which is proposed to mediate the terminal enzymatic step within the pathway. Nevertheless, the results obtained from the present experiments targeting *CYP71AM1* lend further support to the hypothesis that CYP71AM1 is one of the enzymes participating in the biosynthesis of sorgoleone *in vivo*. In addition, as the available evidence suggests that sorgoleone accounts for many of the allelopathic properties attributed to *Sorghum* spp. (e.g. Netzly & Butler, 1986; Einhellig & Souza, 1992; Czarnota *et al*., 2001), experiments are currently being developed to utilize the transformants generated for the present work to examine the effects of RNAi‐mediated inhibition of *CYP71AM1* on the allelopathic potential of *S. bicolor* plants.

*Sorghum bicolor* CYP71AM1 is a member of the CYP71 family, which first appeared in angiosperms and represents the largest known family of plant‐specific P450 enzymes. For example, 84 of the 343 P450 genes found within the japonica rice (cv Nipponbare) genome encode CYP71 members (Nelson & Werck‐Reichhart, 2011). The CYP71 family includes enzymes exhibiting diverse activities associated with the biosynthesis and modification of mono‐, di‐ and sesquiterpenoids, as well as indolic derivatives, cyanogenic glucosides, flavonoids, aldoximes and nitriles (Nelson & Werck‐Reichhart, 2011; Hamberger & Bak, 2013; Xu *et al*., 2015). The functionally characterized *S. bicolor* enzymes within this family include CYP71E1 (Fig. 6) which, together with CYP79A1, catalyzes the conversion of tyrosine to the aglycone of the cyanogenic glucoside dhurrin (Bak *et al*., 1998). Our phylogenetic analyses placed the *S. bicolor* CYP71AM1 sequence within a strongly supported clade containing both CYP71AM and CYP71D subfamily members (Fig. 6). Interestingly, all of the plant species represented within this clade are either known to produce alkylresorcinols or, as is the case for *Aegilops tauschii*, are progenitors to an alkylresorcinol‐producing species (*Triticum aestivum*); therefore, it will be of particular interest to examine the activities of additional CYP71AM/CYP71D subfamily members in future work, as this clade may represent P450 enzymes which modify alkyresorcinols or other classes of phenolic lipids, such as alkylphenols, anacardic acids and alkylcatechols (Kozubek & Tyman, 2005).

The *S. bicolor* CYP71AM1 sequence will undoubtedly serve as a valuable tool for the analysis of phenolic lipid biosynthetic pathways from other plants, and its inclusion within CYP71 expands the known repertoire of activities associated with this enzyme family. Furthermore, as *S. bicolor* CYP71AM1 probably represents the enzyme responsible for the production of dihydrosorgoleone from the sorgoleone pathway intermediate 5‐pentadecatrienyl resorcinol‐3‐methyl ether *in vivo*, genes encoding enzymes catalyzing all of the biosynthetic steps leading to the biosynthesis of dihydrosorgoleone, beginning with palmitoleoyl‐CoA, have now been identified (Dayan *et al*., 2003; Pan *et al*., 2007; Baerson *et al*., 2008; Cook *et al*., 2010). Thus, the present work provides new genetic engineering opportunities in plants, not only for the alteration of sorgoleone content, potentially leading to the development of transgenic crops with reduced reliance on synthetic herbicides, but also for the use of plant cells as bioreactors to provide an efficient means of production of sorgoleone and related phenolic lipids on a large scale.

## Author contributions

Z.P., S.R.B., I.K. and S.O.D. designed the study; Z.P., S.R.B., M.W., J.B‐H., A.M.R., M.E.F. and F.E.D. performed all the experiments; N.P.D.N. performed chemical synthesis; B.P.N. and X.W. performed phylogenetic tree and modeling analysis; Z.P. and S.R.B. analyzed the data and wrote the manuscript.

## Supporting information

Please note: Wiley Blackwell are not responsible for the content or functionality of any Supporting Information supplied by the authors. Any queries (other than missing material) should be directed to the *New Phytologist* Central Office.

**Fig. S1** Amino acid sequence alignment of *Sorghum bicolor* P450s.
**Fig. S2** Carbon monoxide difference spectra of sorghum P450 clones.
**Fig. S3** Transient expression of CYP71AM1 in *Nicotiana benthamiana* as detected by quantitative reverse transcription‐polymerase chain reaction (RT‐qPCR).
**Fig. S4** Binary vector used for RNA interference (RNAi)‐mediated inhibition of *CYP71AM1* expression in *Sorghum bicolor*.
**Table S1** Primers used for quantitative reverse transcription‐polymerase chain reaction (RT‐qPCR) assays and preparation of plasmid constructs
**Table S2** Taxon, accession number and known function for cytochrome P450 protein sequences used in phylogenetic analyses
