# Supplementary material for: A cytochrome P450 CYP71 enzyme expressed in Sorghum bicolor root hair cells participates in the biosynthesis of the benzoquinone allelochemical sorgoleone
Source: New Phytol. 2018 Feb 20;218(2):616–29. doi: 10.1111/nph.15037 (PMC5887931; doi:10.1111/nph.15037)
Supplement: Supplementary file 1 — Fig. S1 Amino acid sequence alignment of Sorghum bicolor P450s. Fig. S2 Carbon monoxide difference spectra of sorghum P450 clones. Fig. S3 Transient expression of CYP71AM1 in Nicotiana benthamiana as detected by quantitative reverse transcription‐polymerase chain reaction (RT‐qPCR). Fig. S4 Binary vector used for RNA interference (RNAi)‐mediated inhibition of CYP71AM1 expression in Sorghum bicolor. Table S1 Primers used for quantitative reverse transcription‐polymerase chain reaction (RT‐qPCR) assays and preparation of plasmid constructs Table S2 Taxon, accession number and known function for cytochrome P450 protein sequences used in phylogenetic analyses [file NPH-218-616-s001.pdf]

***New Phytologist* Supporting Information Figs S1–S4 and Tables S1 & S2**

Article title: A cytochrome P450 CYP71 enzyme expressed in *Sorghum bicolor* root hair cells participates in the biosynthesis of the benzoquinone allelochemical sorgoleone

Authors: Zhiqiang Pan, Scott R. Baerson, Mei Wang, Joanna Bajsa-Hirschel, Agnes M. Rimando, Xiaoqiang Wang, N.P. Dhammika Nanayakkara, Brice P. Noonan, Michael E. Fromm, Franck E. Dayan, Ikhlas A. Khan, Stephen O. Duke

Article acceptance date: 08 January 2018

The following Supporting Information is available for this article:

Fig. S1 Amino acid sequence alignment of *Sorghum bicolor* P450s.
Fig. S2 Carbon monoxide difference spectra of sorghum P450 clones.
Fig. S3 Transient expression of CYP71AM1 in *Nicotiana benthamiana* as detected by RT-qPCR.
Fig. S4 Binary vector used for RNAi-mediated inhibition of *CYP71AM1* expression in *Sorghum bicolor*.
Table S1 Primers used for RT-qPCR assays and preparation of plasmid constructs.
Table S2 Taxon, accession number, and known function for cytochrome P450 protein sequences used in phylogenetic analyses.

Fig. S1 Amino acid sequence alignment of *Sorghum bicolor* P450s. The alignment was performed by using the ClustalW method in MegAlign (DNASTAR, Version 10.1.1). Conserved sequence motifs are highlighted.

```
CYP71AM1  MDEYFVDLPYPNLCLYGSCLVLAVVVARAIILSGSGKKPGGLPPGPWQLPVIGSLHHLLR  60
CYP71AF1  MDQEL-QSSYHY-LFWAAILLLPL-LAINVKL-RRRNHGNKPPPGPWQLPVIGSLHHLVG  56
```

Proline-rich region
(P/I)PGXP(G/P)XP

```
CYP71AM1  GLPHHAIRDLSLRHGPLMLLRICERTAIVVSSAEAVAEMLKRHDAAFSERPSSPGIEELS  120
CYP71AF1  ALPHRAMRDLARRHGPLMLLRLGELPVVVASSPDAAREVMRTHDAAFATRPRTATLRELT  116

CYP71AM1  RHGQGVIFAPYGDHWRLLRRILMTELLSPRRVEAFRHIREDEAARLVSSLSSL--PQPVD  178
CYP71AF1  RDGLGVAFAPHGEHWRQLRKLCVTELLSARRVRSLRGGREAEAANLVASVASSSSSKAVN  176

CYP71AM1  MDERLEVFVADSSVRAILGDRLPDRAAFLKMVKAGQDPSSLFDLRDLFPSSWLVRMLPR-  237
CYP71AF1  VSALLATYVTDAVVRAVVGGQIRDRDAFLEKLDEGVRVAAGFSLADVFPSSRLARAFSGA  236

CYP71AM1  SRKAERHLQEMFRLMDDILVSHSQRRVDDDSPDGGGGGAVDEEHDMVDVLLRIQKQGDMR  297
CYP71AF1  ARAAEAHHREMTRLMDGVIAEHQERRA---------AGAGNDEDDLLDVLLRIQKHGGLQ  287

CYP71AM1  VSLNHGVIRAALIDAVGAALDTTSTTLRWAMAELIANPRVMHKAQLEIRRVMAAGQQRRV  357
CYP71AF1  VPLDMGTIRAVIIDLFSAGSETTATTLQWAMAELMRHPAALRKAQAEVRRVLAG--QNRV  345
```

O_2-binding region
(A/G)GX(D/E)T(T/S)

```
CYP71AM1  HEATLRDLHYLKAVIKETLRLHPPAPF-VPRVCLDDGIKIQGYHVPRGTIVVANVWAISR  416
CYP71AF1  AEDALPKMHYLQLVIKETLRLHAAVPLLLPRECQEETRGVLRYDVPRGAMVLVNAWAIGR  405

CYP71AM1  DPKYWE-DPDMFIPERFHQGDPDHHRCFDFKGFDFEFTPFGAGRRMCPGMNFAHMNVEIA  475
CYP71AF1  DAASWGPDAEEFRPERFEDGGAR--AKVDFRGTDFEFVPFGAGRRICPGIALGLAVMELG  463
```

E-R-R triade

Heme binding
PFGXGRRXCXG

```
CYP71AM1  LASLLYHFDWKLPDGATPEEIDMTELWGVTVARKAKLLLHPIPCIPAAASIDA   528
CYP71AF1  LASLLFHFDWALPGGAAPEELDMAEGLGITARRKNDLWLQATVRVTVPIV---   513
```

Fig. S2 Carbon monoxide difference spectra of sorghum P450 clones. The $Fe^{2+}\cdot CO$ versus Fe^{2+} spectra were measured on microsomes extracted from yeast cells expressing CYP71AM1 (a) and CYP71AF1(b) with empty vector as control (shown in red).

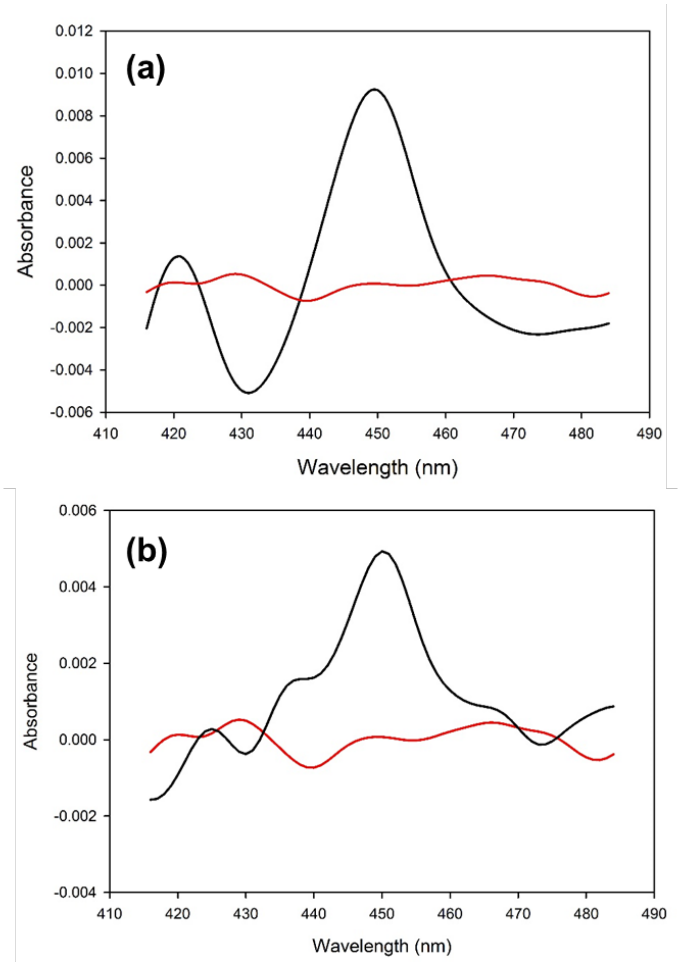

Fig. S3 Transient expression of CYP71AM1 in *Nicotiana benthamiana* as detected by RT-qPCR. Three leaves from each plant and nine plants for each time point were collected and flash-frozen in liquid nitrogen. Total RNA for these experiments was isolated from *N. benthamiana* leaves infiltrated with an *Arobacterium tumefaciens* strain harboring the binary expression vector pLHG using an RNeasy plant mini kit (Qiagen) according to the manufacturer's instructions. RNAs were then treated with RNase-free DNase I to remove residual DNA contamination and re-purified with an RNeasy MinElute Cleanup Kit (Qiagen) according to the manufacturer's instructions. The quality and quantity of prepared total RNAs were assessed according to the MIQE Guidelines (Bustin *et al.*, 2009; Bustin *et al.*, 2010). RT-qPCR was performed in triplicate using a CFX96 Touch™ Real-Time PCR Detection System (Bio-Rad). The RT-qPCR reactions were conducted using iTaq Universal SYBR Green Supermix (Bio-Rad) according to the manufacturer's instructions. The primers used for each gene are provided in Table S1. The relative expression levels were calculated using Bio-Rad CFX Manager software (version 3.1). All values were normalized to the expression values of two reference genes (*N. benthamiana* Actin and PP2A) according to Liu *et al*. (Liu *et al.*, 2012).

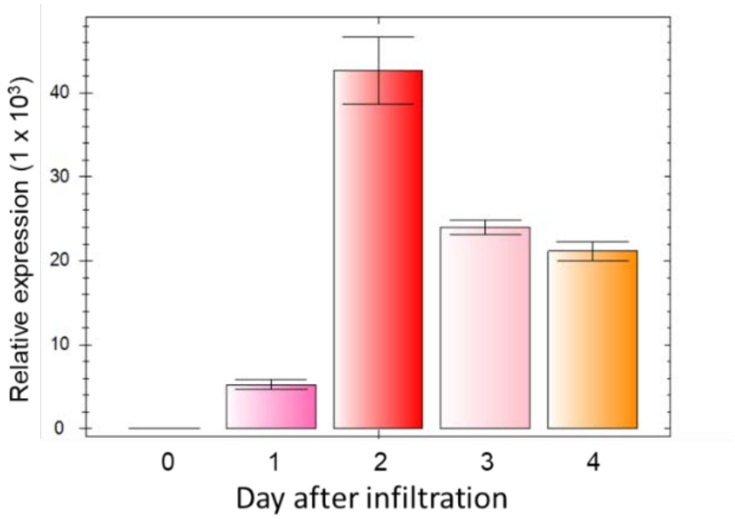

Fig. S4 Binary vector used for RNAi-mediated inhibition of *CYP71AM1* expression in *Sorghum bicolor*. The construction of pCYP-RNAi was carried out as described previously (Cook *et al.*, 2010). The sense and antisense of CYP71AM1 target region were separated by intron 1 of the Arabidopsis FAD2 gene (FAD2 int). The transcription of this cassette was driven by the constitutive *Zea mays* polyubiquitin-1 gene promoter and intron 1 (P-Ubi-Zm). To facilitate the selection of transformants, a GFP/NPTII expression cassette was also inserted within the T-DNA region.

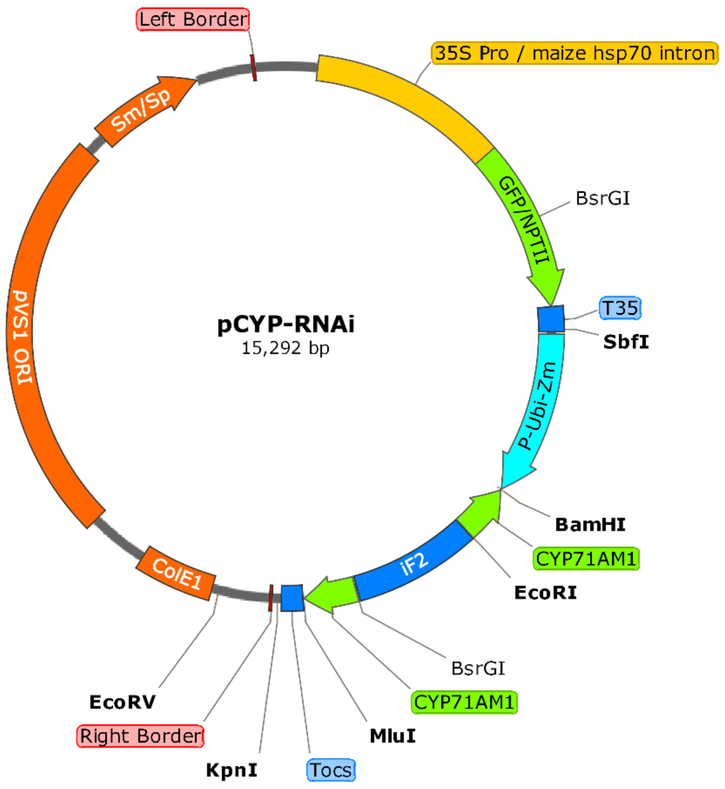

Table S1 Primers used for RT-qPCR assays and preparation of plasmid constructs.

Primer name	Sequence (5' to 3')	Description
**RT-qPCR experiments**		
21G12F	AAGATCCAAGGCTACCATGTGC	Forward for CYP71AM1
21G12R	AACGTTGGCGACGACTATTG	Reverse for CYP71AM1
71F08F	GGCGAGGAGGAAGAACGAC	Forward for CYP71AF1
71F08R	GAACAGTAACGCGTACGGTGG	Reverse for CYP71AF1
18SF	GGCTCGAAGACGATCAGATACC	18s rRNA
18SR	TCGGCATCGTTTATGGTT	18s rRNA
**Yeast expression constructs**		
ZP16	GGGTACCATGGACGAATACTTTGTTGACCTGC	5' for ORF of CYP71AM1 with KpnI for pYeDP60
ZP17	GGAATTCGTGTGAATGGAAATATTTTATGCATCAA	3' for ORF of CYP71AM1 with EcoRI
ZP71	CGGTACCATCATGGACCAAGAACTCCAAT	5' for ORF of CYP71AF1 with KpnI for pYeDP60
ZP72	GGAATTCTGAAACTTTCGTAAAACCTCCA	3' for ORF of CYP71AF1 with EcoRI
***N. benthamiana* expression construct**		
ZP812	cctggccatggcggccGCCTCTAGAGTCGACCTGCAGG	2xP35S 5' with NotI-SfiB
ZP768	CGTCCATTGTTTAccgttcgtaaatggtgaaaatt	2xP35S 3' with CYP71AM1 5' start codon
ZP769	caccatttacgaacggTAAACAATGGACGAATACTTTGTTGACC	CYP71AM1 5' with 2xP35S 3'
ZP823	ccaggcccttaaggccACTAGTaggtttgacctgcacttcatttgg	Ocs 3' with SpeI-SfiA
**RNA interference construct**		
ZP725	CCTGAATTCTTACGACTGCACCCTCC	CYP71AM1 5' with EcoRI for RNAi
ZP726	TCTGGATCCGTGTGAATGGAAATATTTTATGCATC	CYP71AM1 3' with BamHI for RNAi
ZP727	CCTTGTACACTTACGACTGCACCCTCC	CYP71AM1 5' with BsrGI for RNAi
ZP728	TCTACGCGTGAATGGAAATATTTTATGCATC	CYP71AM1 3' with MluI for RNAi

Table S2 Taxon, accession number, and known function for cytochrome P450 protein sequences used in phylogenetic analyses.

**Species**	**P450**	**Accession**	**Function**
Aegilops tauschii	CYP71D8	XP_020186750	
Aegilops tauschii	CYP71D10	XP_020186752	
Aegilops tauschii	CYP71D11	XP_020151284	
Ammi majus	CYP71AJ1	Q6QNI4	psoralen synthase
Arabidopsis thaliana	CYP71B15	Q9LW27	bifunctional dihydrocamalexate synthase/camalexin synthase
Arabidopsis thaliana	CYP71A13	O49342	indoleacetaldoxime dehydratase
Artemisia annua	CYP71AV1	Q1PS23	amorpha-4,11-diene 12-monooxygenase
Brachypodium distachyon	CYP71AM1	KQK13613	
Brachypodium distachyon	CYP71AF5	KQJ84528	
Brachypodium distachyon	CYP71AF6	KQJ84527	
Catharanthus roseus	CYP71D12	ACM92061	tabersonine-16-hydroxylase (T16H)
Catharanthus roseus	CYP71D351	U5HKE8	tabersonine 16-hydroxylase 2 (T16H2)
Catharanthus roseus	CYP71BJ1	ADZ48681	tabersonine/lochnericine 19-hydroxylase
Cichorium intybus	CYP71BL3	G3GBK0	costunolide synthase (CiCOS)
Cichorium intybus	CYP71AV8	E1B2Z9	(+)-valencene oxidase
Eschscholzia californica	CYP80B1	AAC39453	(S)-N-methylcoclaurine 3'-hydroxylase
Glycine max	CYP71A10	AF022157	catalyzed the metabolism of phenylurea herbicides
Helianthus annuus	CYP71BL1	AEI59779	GAA 8β-hydroxylase (HaG8H)
Hyoscyamus muticus	CYP71D55	ABS00393	premnaspirodiene oxidase (HPO)
Lactuca sativa	CYP71BL2	AEI59780	costunolide synthase (LsCOS)
Manihot esculenta	CYP71E7	AY217351	2-methylbutanal oxime monooxygenase
Mentha gracilis	CYP71D95	Q6WKY9	limonene-3-hydroxylase
Mentha piperata	CYP71D13	AY281027	limonene-3-hydroxylase
Mentha spicate	CYP71D18	Q9XHE8	limonene-6-hydroxylase
Nicotiana tabacum	CYP71D16	AAD47832	terpene hydroxylase
Nicotiana tabacum	CYP71D20	AF368376	5-epi-aristolochene hydroxylase (EAH)
Oryza sativa	CYP71Z6	A3A871	ent-isokaurene C2-hydroxylase
Oryza sativa	CYP71Z7	Q6YV88	ent-cassadiene C2-hydroxylase
Oryza sativa	CYP71D8	XP_015626856	
Oryza sativa	CYP71D10	XP_015624052	
Oryza sativa	CYP71AF1	XP_015643928	
Populus trichocarpa	CYP71B40v3	AIU56748	converts various aldoximes to corresponding nitriles *in vitro*
Populus trichocarpa	CYP71B41v2	AIU56747	converts various aldoximes to corresponding nitriles *in vitro*
Populus trichocarpa	CYP71B63v2	AIU56749	
Prunus mume	CYP71AN24	AB920492	phenylacetaldehyde oxime monooxygenase

Setaria italica	CYP71AM1	XP_004954907	
Setaria italica	CYP71AM5	Setaital1.21424	
Sorghum bicolor	CYP71E1	AF029858	4-hydroxyphenylacetaldehyde oxime monooxygenase
Sorghum bicolor	CYP71AF1	MG020490	
Sorghum bicolor	CYP71AM1	MG020489	pentadecatrienyl resorcinol hydroxylase, present study
Triticum urartu	CYP71D7	EMS60990	
Vitis vinifera	CYP71BE5	BAT70338	alpha-guaine 2-oxidase (VvSTO2)
Zea mays	CYP71C36	ACG46235	
Zea mays	CYP71W7	NP_001288388	
Zea mays	CYP71Y10	ACG29861	
Zea mays	CYP71K15	ACG41433	
Zea mays	CYP71D8	XP_ 008680404	
Zingiber zerumbet	CYP71BA1	BAJ39893	alpha-humulene 10-hydroxylase

References

Bustin SA, Beaulieu JF, Huggett J, Jaggi R, Kibenge FS, Olsvik PA, Penning LC, Toegel S. 2010. MIQE precis: Practical implementation of minimum standard guidelines for fluorescence-based quantitative real-time PCR experiments. *BMC Molecular Biology* **11**: 74.

Bustin SA, Benes V, Garson JA, Hellemans J, Huggett J, Kubista M, Mueller R, Nolan T, Pfaffl MW, Shipley GL, et al. 2009. The MIQE guidelines: minimum information for publication of quantitative real-time PCR experiments. *Clinical Chemistry* **55**: 611-622.

Cook D, Rimando AM, Clemente TE, Schroder J, Dayan FE, Nanayakkara NP, Pan Z, Noonan BP, Fishbein M, Abe I, et al. 2010. Alkylresorcinol synthases expressed in *Sorghum* bicolor root hairs play an essential role in the biosynthesis of the allelopathic benzoquinone sorgoleone. *Plant Cell* **22**: 867-887.

Liu D, Shi L, Han C, Yu J, Li D, Zhang Y. 2012. Validation of reference genes for gene expression studies in virus-infected *Nicotiana benthamiana* using quantitative real-time PCR. PLoS One **7**: e46451.
